# Analysis of DRG remuneration of acute fracture procedures versus elective total arthroplasty for the German health care system – Is elective total arthroplasty really more cost-effective?

**DOI:** 10.1007/s00068-026-03173-w

**Published:** 2026-04-21

**Authors:** Katja Hierl, Maximilian Kerschbaum, Florian Baumann, Volker Alt

**Affiliations:** Department of Trauma Surgery, University Hospital Regensburg, Franz-Josef-Strauß-Allee 11, 93053 Regensburg, Germany

**Keywords:** aG-DRG system, Cost efficiency, Traumatology, Elective orthopaedics, Hip, Shoulder

## Abstract

**Purpose:**

Elective arthroplasty is considered a cost-effective procedure compared to trauma-related surgical interventions. This study aims to compare remuneration, certain ratios regarding implant costs, operation (OR) time and length of stay (LOS) as well as contribution margins including personnel costs for acute fracture interventions and elective arthroplasty procedures at the hip and shoulder joint in the aG-DRG system.

**Methods:**

Based on billing data of 212 inpatient treatment cases from a University Orthopaedic Trauma Department reimbursements of nail fixation and hemi-arthroplasty (HA) for proximal femoral fractures versus elective total hip arthroplasty (THA) for osteoarthritis were analysed. Locking plate fixation and reverse total shoulder arthroplasty (rTSA) for proximal humeral fractures were compared to elective rTSA for osteoarthritis. Different ratios of the DRG remuneration in relation to LOS, OR time and implant costs but also personnel costs and contribution margins were calculated.

**Results:**

DRG reimbursement was highest for fracture arthroplasty of the shoulder (9.050 EUR, rTSA) and the hip (6.930 EUR, HA). Regarding LOS, DRG reimbursement was highest for elective rTSA (1.641 EUR/day). Nail fixation of proximal femoral fractures showed the highest DRG reimbursement regarding OR time (111 EUR/min) and implant costs (15 EUR/EUR). The contribution margin was highest for fracture rTSA (6.339 EUR) and lowest for elective THA (3.842 EUR).

**Conclusion:**

Elective total hip and shoulder arthroplasty is not generally more cost-effective in the aG-DRG system. Acute fracture-related interventions seem economically more advantageous with regard to implant costs and OR time, elective total arthoplasty seems more profitable regarding LOS in the hospital.

## Introduction

In Germany, reimbursement of inpatient treatments is regulated via DRG (Diagnosis Related Groups) system, which has been updated in 2020 to the aG-DRG system with outsourcing of nursing care costs from the DRG flat rate per case. The DRG remuneration comprises all medical costs, such as implant or drug costs, and reflects the average treatment costs of inpatient hospital stays [1]. This has led to increasing economic pressure on hospitals with a rise in the number of profitable treatment cases and a reduction in the length of inpatient stays [2–4] due to the revenue- and cost-oriented incentives of the DRG system. For example, elective hip arthroplasty is considered a cost-effective treatment, even in older patients, with early surgical intervention and a lower preoperative WOMAC score leading to better economic outcomes [5–8]. In contrast, the treatment of patients with severe comorbidities and longer length of stay, such as geriatric patients with hip fractures, seems not cost covering for the hospitals due to the higher treatment costs [9–11].

In acute traumatology and elective orthopaedics, there is a certain overlap of surgical interventions, which are comparable from a medical and economic perspective. For example, elective total hip arthroplasty (THA) for osteoarthritis is similar to acute hemi-arthroplasty (HA) or nail fixation of proximal femoral fractures regarding the type of surgical procedure as well as the cost and revenue structure.

It is so far unclear, whether there is a comparable DRG reimbursement between similar traumatological and elective orthopaedic surgical interventions or not. This uncertainty refers to the overall DRG reimbursement but also to relevant ratios, such as DRG remuneration per length of stay (LOS), per operation (OR) time and per implant costs. This study aims to conduct a cost-revenue analysis of standard orthopaedic and trauma surgical procedures at the proximal femur and the proximal humerus, as no current data is available for this.

With regard to LOS, OR time, implant costs and personnel costs, the DRG revenue structure of trauma and orthopaedic hip and shoulder surgeries will be compared and the contribution margins determined. The question arises, whether relevant differences in reimbursement exist between acute trauma surgery and elective orthopaedic procedures in the same anatomical region, which could create certain false incentives.

## Materials and methods

### Data collection

Data of all inpatient treatment cases from a University Orthopaedic Trauma Department treated for acute fractures or elective arthroplasty procedures of the hip and shoulder joint between January 2020 and December 2022 were collected retrospectively. All cases with Clinical Complexity Level (CCL) relevant secondary diagnoses and higher-ranked DRGs, as well as cases discharged by other departments of the University Hospital were excluded. Based on the ICD and OPS codes of the diagnoses and surgical procedures, as well as the DRG classification the treatment cases were filtered from the dataset of the Medical Controlling Department of the University Hospital. Clinical data and implant costs were collected from clinical documents (medical reports) as well as surgical and implant documentation from the hospital information system (SAP IS-H, Industrial Solutions Hardware, Walldorf, Germany). Cost data for the implants used were provided by the Purchasing and Logistics Department of the University Hospital. Three groups were compared at the hip and shoulder:

### Hip

#### Group 1

DRG I08F (Fig. 1).

- Main diagnosis: Per- or subtrochanteric femoral fracture.
- Surgical intervention: Nail fixation (Gamma nail).

#### Group 2

DRG I47B (Fig. 2).

- Main diagnosis: Medial or lateral femoral neck fracture.
- Surgical intervention: Cemented hemi-arthroplasty (HA).

#### Group 3

DRG I47C (Fig. 3).

- Main diagnosis: Primary or secondary hip osteoarthritis.
- Surgical intervention: Cementless total arthroplasty (TA).

**Fig. 1:**
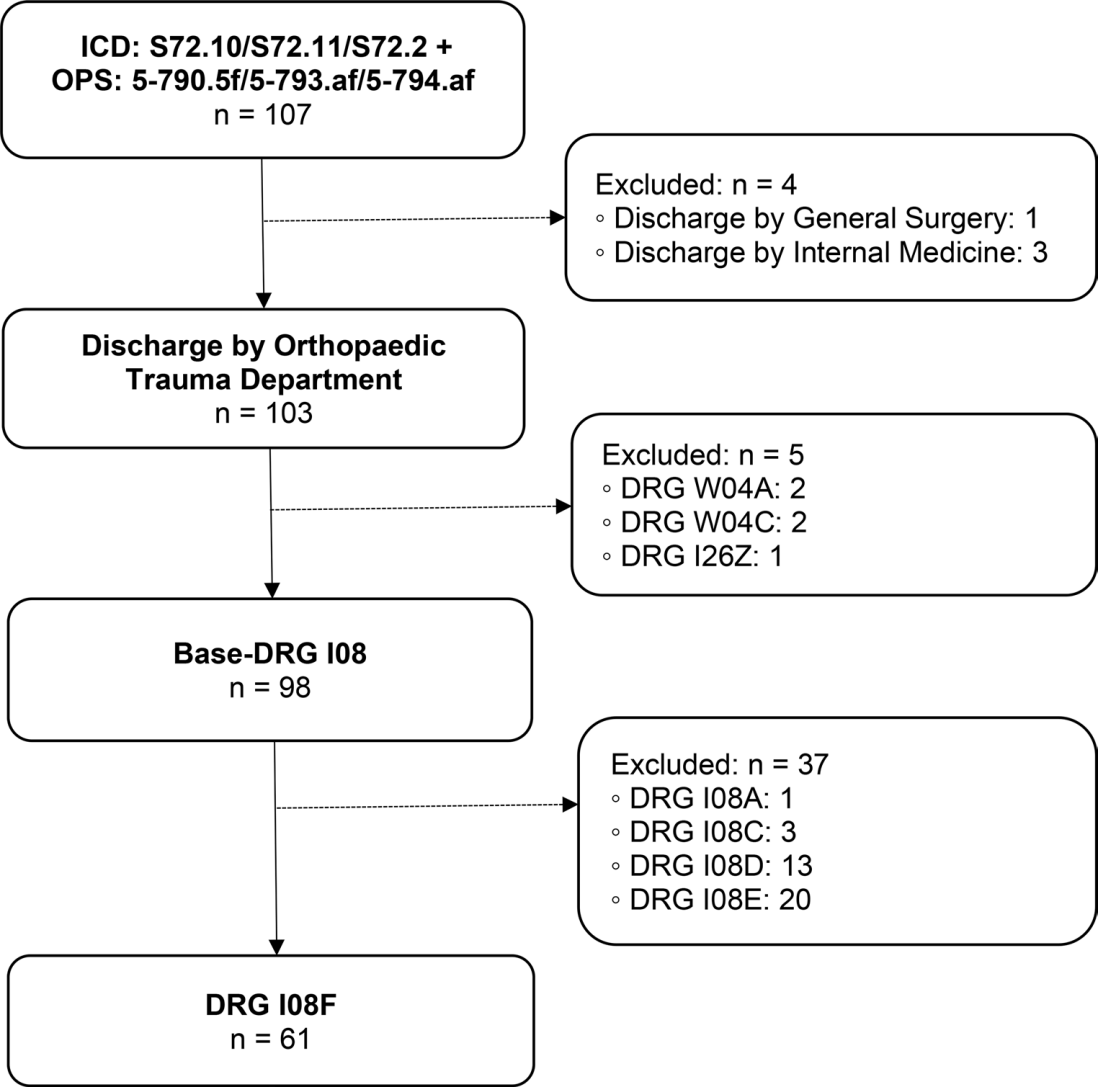
Group 1 flow chart: Proximal femoral fracture with nail fixation. n = number of cases

**Fig. 2:**
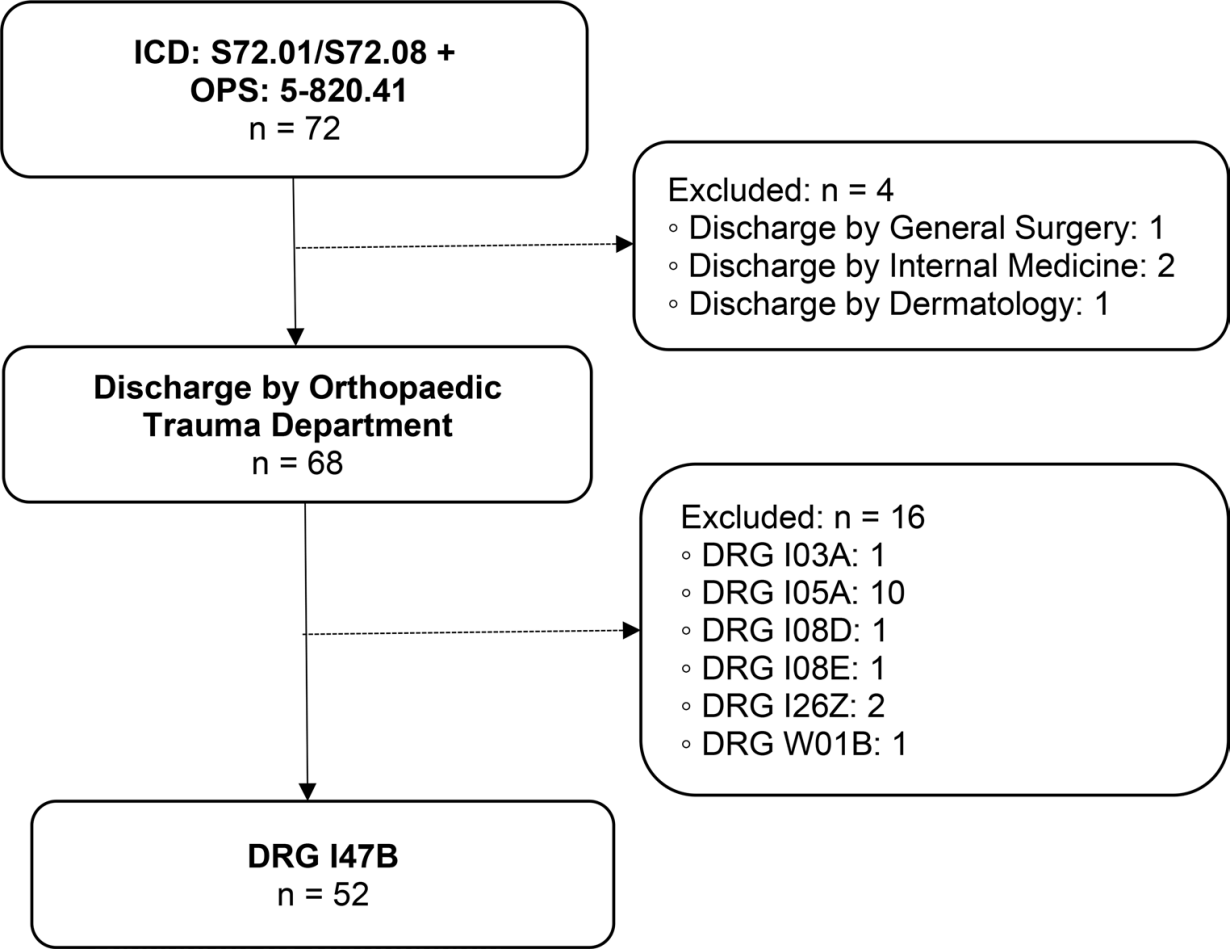
Group 2 flow chart: Femoral neck fracture with cemented HA. n = number of cases

**Fig. 3:**
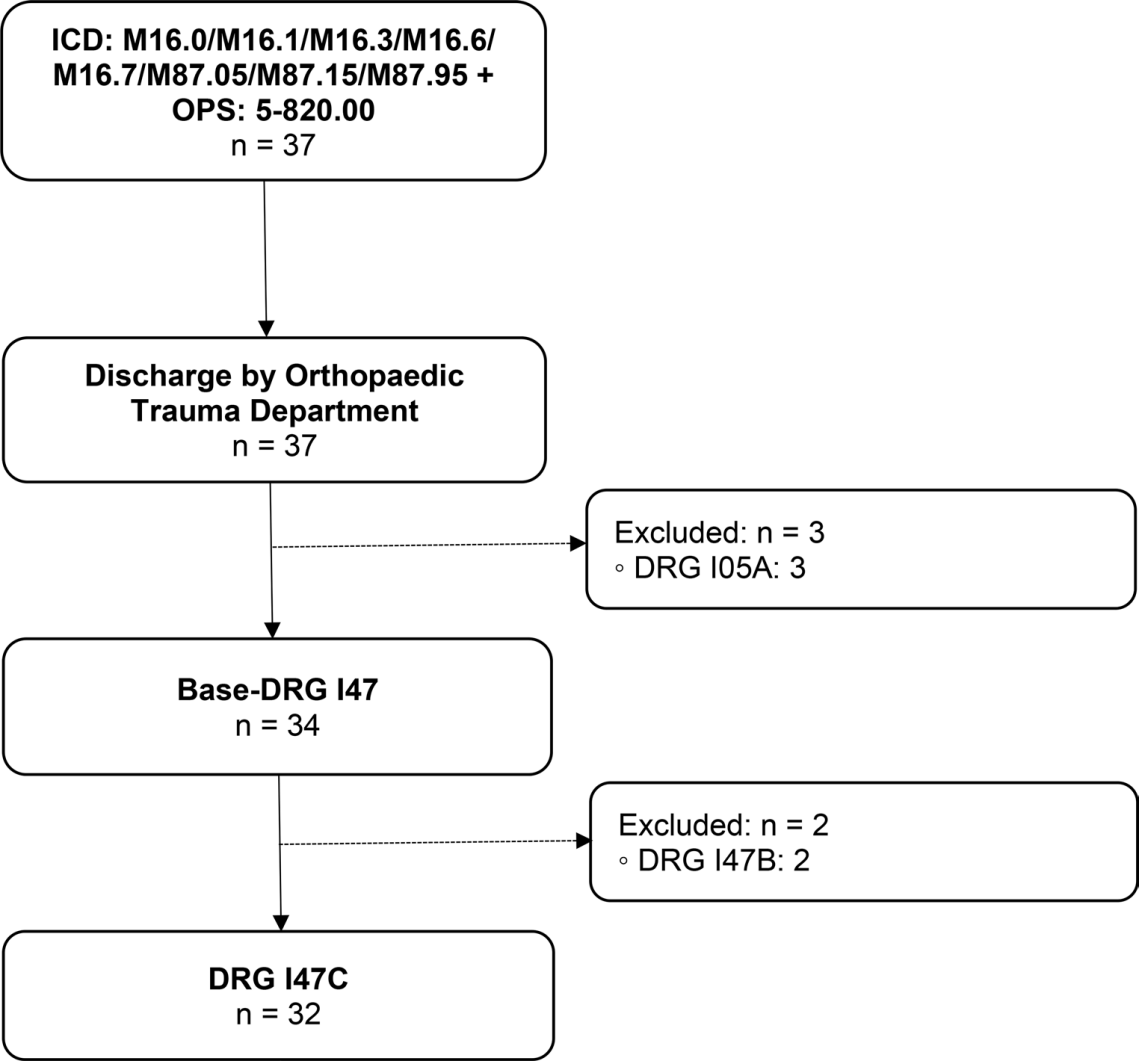
Group 3 flow chart: Hip osteoarthritis with cementless TA. n = number of cases

### Shoulder

#### Group 4

DRG I13E (Fig. 4).

- Main diagnosis: Proximal humeral fracture.
- Surgical intervention: Locking plate fixation (PHILOS plate).

#### Group 5

DRG I05B (Fig. 5).

- Main diagnosis: Proximal humeral fracture.
- Surgical intervention: Reverse total shoulder arthroplasty (rTSA).

#### Group 6

DRG I05B (Fig. 6).

- Main diagnosis: Primary or secondary shoulder osteoarthritis.
- Surgical intervention: Reverse total shoulder arthroplasty (rTSA).

**Fig. 4:**
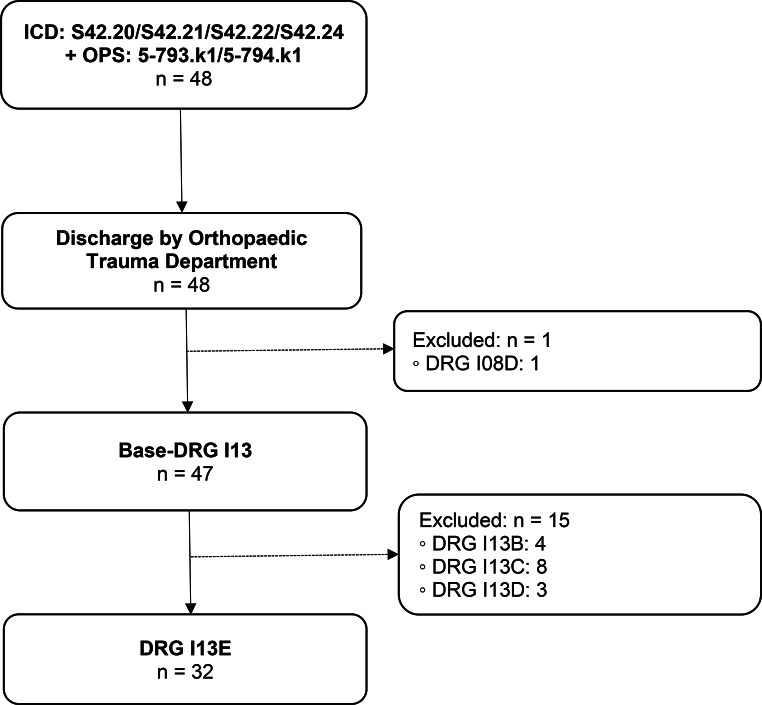
Group 4 flow chart: Proximal humeral fracture with locking plate fixation. n = number of cases

**Fig. 5:**
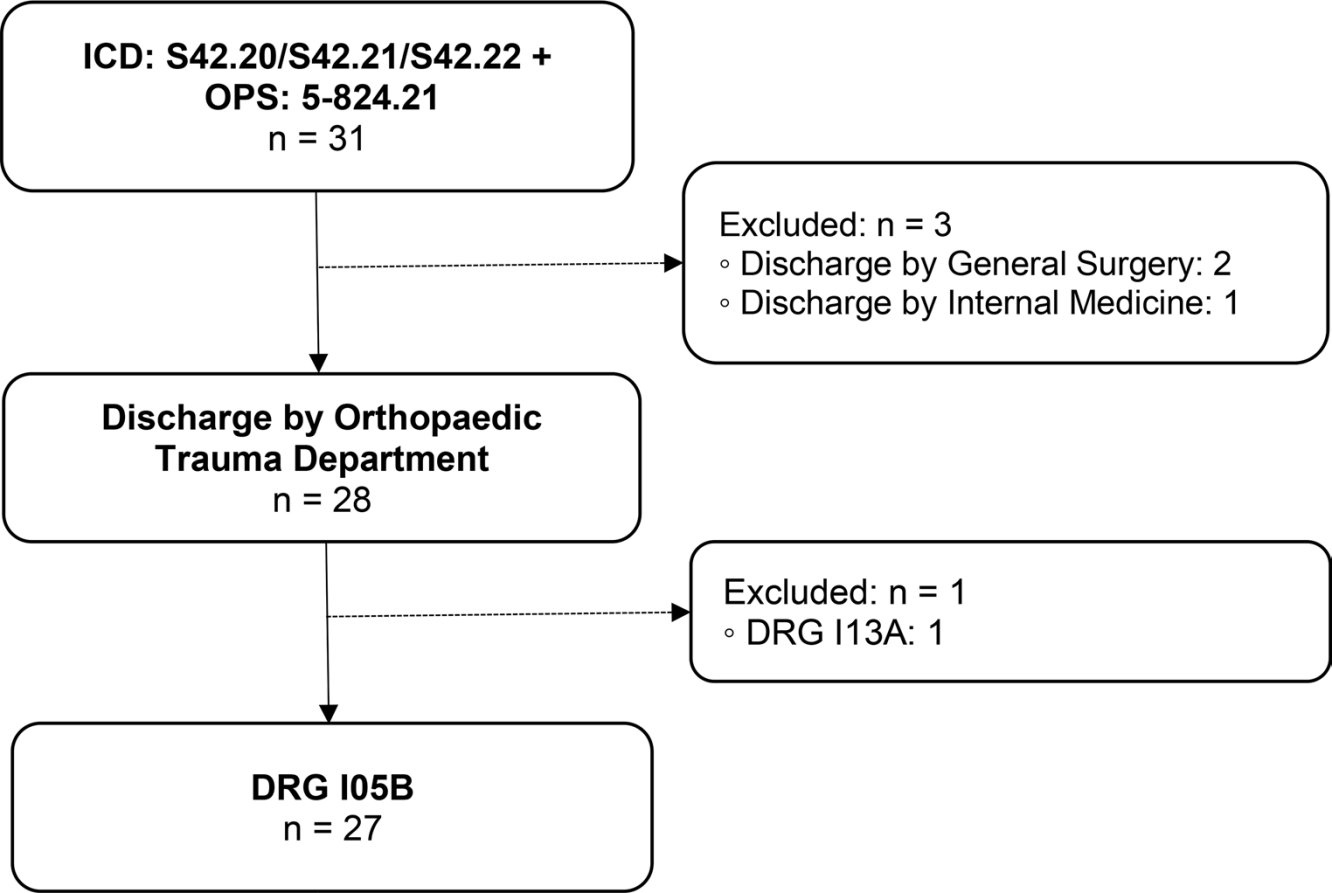
Group 5 flow chart: Proximal humeral fracture with rTSA. n = number of cases

**Fig. 6:**
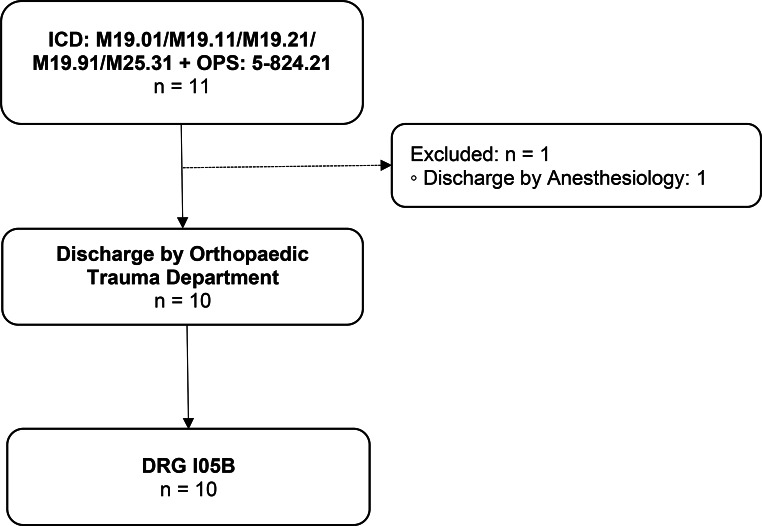
Group 6 flow chart: Shoulder osteoarthritis with rTSA. n = number of cases

### Statistical analysis

Data were collected in tabular form using Excel Version 16 (Microsoft Corporation, Redmond, USA) and pseudonymized by assigning case and patient identification numbers. The research project received prior approval from the ethics committee of the affiliated university (Nr. 23-3354-104).

### Clinical data

- Gender (female/male).
- Age (years).
- LOS total (days).
- LOS peripheral ward (days).
- LOS Intensive Care Unit (ICU, days).
- Operation (OR) time (minutes).

### Billing data

- DRG
- Patient Clinical Complexity Level (PCCL).
- Deduction (Euro, EUR).
- Surcharge (EUR).
- Effective Cost Weight (CW).
- Effective DRG remuneration (EUR).
- Additional charge (EUR).
- Total remuneration (EUR).
- State base case value Bavaria (EUR).
- Nursing care remuneration value (EUR).
- Implant costs (EUR).

As descriptive statistic mean values and standard deviations (SD) of the clinical and billing parameters were calculated using Excel 16 (Microsoft Corporation, Redmond, USA). DRG classification of the cases was performed using the 3 M™ Kodip^®^ Suite grouper software (3 M Company, Neuss, Germany) versions 2020 to 2022. The DRG revenues were based on the state base case values for Bavaria 2020 to 2022, the nursing care revenues on the preliminary or hospital-specific nursing care remuneration values.

### Calculation of ratios

To evaluate the relationship between DRG reimbursement and LOS, OR time and implant costs the following ratios were calculated for group 1–6:

$$\:\boldsymbol{R}\boldsymbol{a}\boldsymbol{t}\boldsymbol{i}\boldsymbol{o}\:1=\:\frac{DRG\:reimbursement\:\left(EUR\right)}{LOS\:\left(day\right)}$$

$$\:\boldsymbol{R}\boldsymbol{a}\boldsymbol{t}\boldsymbol{i}\boldsymbol{o}\:2=\:\frac{DRG\:reimbursement\:\left(EUR\right)}{OR\:time\:\left(minute\right)}$$

$$\:\boldsymbol{R}\boldsymbol{a}\boldsymbol{t}\boldsymbol{i}\boldsymbol{o}\:3=\:\frac{DRG\:reimbursement\:\left(EUR\right)}{implant\:costs\:\left(EUR\right)}$$

### Calculation of costs and contribution margins

#### Personnel costs

For the methodology of personnel cost calculations for operating room, ICU and peripheral ward, reference is made to a previous study on a health-economic model for personnel cost calculation in a University Orthopaedic Trauma Department [12].

In summary, the calculations were based on the remuneration for each classification, as specified in the collective agreement (TV-L and TV-Doctors University Hospitals, 2024) for the respective professional groups (medical service, nursing service). In addition, an employer contribution to social security of 25,59% was applied. The calculations were prepared using Excel Version 16 (Microsoft Corporation, Redmond, USA) and the means as well as standard deviations per group were determined.

Personnel costs incurred outside of medical care, e.g. administration or cleaning, and other cost blocks, such as medication or energy, were not included in the calculations, as we focussed on the costs of personnel directly involved in the medical treatment in the OR and on the ward.

#### Calculation of OR personnel costs

The calculation of OR personnel costs was based on the activity times (in minutes) of all personnel directly involved in the surgery (surgeons, surgical nurses) as recorded in the OR documents for each treatment case. The operation time refers to the incision suture time of the surgical procedures. All operations, both for fracture treatment and elective prosthesis implantation, were performed by surgeons with senior physician qualifications as well as specialist or resident physician qualifications. The operations were either performed by the senior physicians themselves or as training procedures by the specialist or resident physicians under supervision. It was assumed that for all operations, one anesthesiologist and one anesthesiology nurse were present for the entire duration of the surgical procedure, and the corresponding personnel costs per operation were determined.

#### Calculation of personnel costs for ICU and peripheral ward

The personnel costs for ICU and peripheral ward (each 24 beds) were calculated as a model under the assumption of a medical and nursing service deployment that was based on those of a University Hospital and has no universal validity. The calculations were based on a 3-shift model for nursing staff. For the medical staff, a 2-shift model was used in the ICU, a 1-shift model on the peripheral ward. The requirements of the regulation on the determination of minimum nursing staff levels in nursing-sensitive areas in hospitals were observed. According to the PpUGV (Nursing Staff Lower Limits Ordinance), a patient-to-nurse ratio of 10 to 1 (day shift) and 20 to 1 (night shift) was set for the peripheral ward per shift. For intensive care, a ratio of 2 to 1 was applied for the day shift and 3 to 1 for the night shift.

The personnel costs for the nursing and medical service were averaged for a 24-hour day over a 7-day week, with different staffing on Monday to Friday and Saturday to Sunday/public holidays. By dividing by the number of beds the proportional personnel costs per bed/24-hour day were calculated.

#### Surgical costs

For each treatment case of hip and shoulder, the surgical costs were calculated by adding the OR personnel costs and the implant costs. The surgical costs per group were calculated from the average values.

#### Contribution margins

To approximately calculate the contribution margins, a cost-revenue analysis was performed using the mean values per each group:

Contribution margin = Ø Total revenue – (Ø Surgical costs + Ø Total personnel costs).

## Results

### Hip

145 treatment cases were initially included. Of these, 2 cases with elective total hip arthroplasty and hemophilia A were excluded due to the high additional charges for the perioperative administration of coagulation factor VIII. Table 1 provides an overview of the demographic, clinical and DRG data, as well as the implant costs, for the 143 cases included.

#### Group 1

Proximal femoral fracture with nail fixation

A total of 61 cases with an average LOS of 11 days were evaluated. The DRG reimbursement, including deductions and surcharges, was 5.906 EUR which together with the nursing care reimbursement (2.516 EUR) resulted in the total reimbursement of 8.440 EUR. Due to an increased care requirement an additional charge of 18 EUR could be billed. The OR time was 53 min with implant costs of 398 EUR.

#### Group 2

Femoral neck fracture with cemented HA

52 cases were included. The average LOS was 13 days. The DRG reimbursement was highest with 6.930 EUR, also the total reimbursement (9.600 EUR) and the nursing care reimbursement (2.638 EUR) due to the longest LOS. The additional charge of 32 EUR was based on an increased care requirement and dialysis. The OR time was longest with 87 min, the implant costs were 749 EUR.

#### Group 3

Hip osteoarthritis with cementless TA

A total of 30 cases were analysed with the shortest LOS of eight days. The DRG reimbursement was lowest with 5.781 EUR, as well as the nursing care reimbursement with 1.300 EUR and the total reimbursement (7.243 EUR). The additional charge of 161 EUR was based on extracorporeal photopheresis and dialysis. The implant costs were highest with 1.489 EUR, the OR time was 75 min.

**Table 1:** Data of hip interventions

Parameter	Group 1 Proximal femoral fracture (Mean ± SD)	Group 2 Medial/lateral femoral neck fracture (Mean ± SD)	Group 3 Primary/secondary hip osteoarthritis (Mean ± SD)
Number of cases	61	52	30
Surgical intervention	Nail fixation (Gamma nail)	Cemented hemi-arthroplasty (HA)	Cementless total arthroplasty (TA)
ICD Codes	S72.10, S72.11, S72.2	S72.01, S72.08	M16.0, M16.1, M16.3, M16.6, M16.7, M87.05, M87.15, M87.95
OPS Codes	5-790.5f, 5-793.af, 5-794.af	5-820.41	5-820.00
Gender (female, male)	female: 41, male: 20	female: 36, male: 16	female: 12, male: 18
Age (years)	77,93 ± *12*,*15*	83,42 ± *6*,*79*	63,77 ± *12*,*48*
DRG	I08F	I47B	I47C
PCCL	0,84 ± *1*,*15*	1,13 ± *1*,*26*	1,30 ± *1*,*44*
LOS total (days)	11,36 ± *5*,*84*	12,87 ± *5*,*56*	8,00 ± *2*,*76*
LOS peripheral ward (days)	11,18 ± *5*,*57*	12,58 ± *5*,*24*	8,00 ± *2*,*76*
LOS ICU (days)	0,18 ± *0*,*78*	0,29 ± *1*,*28*	0,00 ± *0*,*00*
Deduction (EUR)	22,81 ± *176*,*65*	187,31 ± *528*,*51*	0,00 ± *0*,*00*
Surcharge (EUR)	156,15 ± *446*,*49*	140,81 ± *381*,*02*	14,03 ± *52*,*48*
Effective CW	1,57 ± *0*,*13*	1,85 ± *0*,*18*	1,54 ± *0*,*02*
Effective DRG remuneration (EUR)	5.905,73 ± *492*,*82*	6.930,05 ± *707*,*38*	5.781,29 ± *82*,*41*
Nursing care remuneration (EUR)	2.516,00 ± *1.813*,*46*	2.637,89 ± *1.519*,*02*	1.300,19 ± *808*,*92*
Additional charge (EUR)	18,30 ± *71*,*20*	32,31 ± *144*,*53*	161,06 ± *534*,*79*
Total remuneration (EUR)	8.440,03 ± *2.164*,*48*	9.600,25 ± *1.974*,*09*	7.242,55 ± *1.176*,*21*
Base rate Bavaria (EUR)	3.757,18 ± *68*,*24*	3.753,17 ± *70*,*71*	3.747,39 ± *67*,*07*
Nursing care fee value (EUR)	244,34 ± *98*,*39*	234,06 ± *97*,*89*	218,31 ± *95*,*53*
OR time (minutes)	53,20 ± *20*,*47*	87,04 ± *21*,*29*	75,33 ± *15*,*36*
Implant costs (EUR)	397,88 ± *85*,*07*	748,71 ± *36*,*73*	1.489,09 ± *142*,*27*

*DRG* diagnosis related groups; *EUR* Euro; *HA* hemi-artrhroplasty; *ICU* intensive care unit; *LOS* length of stay; *OR* operation; *PCCL* patient clinical complexity level; *SD* standard deviation; *TA* total arthroplasty; *CW* cost weight

### Shoulder

A total of 69 patients were included. Table 2 presents the demographic, clinical and DRG data, as well as the implant costs.

#### Group 4

Proximal humeral fracture with locking plate fixation

A total of 32 cases were included with an average LOS of seven days. The DRG reimbursement of 5.226 EUR and the total reimbursement (6.663 EUR) including the nursing care reimbursement of 1.437 EUR were lowest. The OR time of 101 min was longest with implant costs of 724 EUR.

#### Group 5

Proximal humeral fracture with rTSA.

27 cases were analysed. The mean LOS of 12 days was longest and the DRG reimbursement was highest with 9.050 EUR. Due to the fracture situation with bony defect of the proximal humerus and modular type of prosthesis used, an additional charge of 1.345 EUR was billed. The total reimbursement of 12.675 EUR was highest including the nursing care reimbursement of 2.280 EUR. The implant costs were highest with 3.470 EUR, the OR time was 95 min.

#### Group 6

Shoulder ostearthritis with rTSA

Ten cases were included. The average LOS of five days was shortest. The DRG reimbursement (8.695 EUR) and the total reimbursement (9.520 EUR) were second highest. The nursing care reimbursement of 825 EUR was lowest due to the shortest LOS. The OR time of 98 min was comparable to group 5. The implant costs of 3.103 EUR were second highest.

**Table 2:** Data of shoulder interventions

Parameter	Group 4 Proximal humeral fracture (Mean ± SD)	Group 5 Proximal humeral fracture (Mean ± SD)	Group 6 Primary/secondary shoulder osteoarthritis (Mean ± SD)
Number of cases	32	27	10
Surgical intevention	Locking plate fixation (PHILOS plate)	Reverse total shoulder arthroplasty	Reverse total shoulder arthroplasty
ICD Codes	S42.20, S42.21, S42.22, S42.24	S42.20, S42.21, S42.22	M19.01, M19.11, M19.21, M19.91, M25.31
OPS Codes	5-793.k1, 5-794.k1	5-824.21	5-824.21
Gender (female, male)	female: 20, male: 12	female: 22, male: 5	female: 8, male: 2
Age (years)	62,19 ± *12*,*23*	72,63 ± *10*,*35*	71,90 ± *8*,*57*
DRG	I13E	I05B	I05B
PCCL	0,31 ± *0*,*77*	1,00 ± *1*,*39*	0,20 ± *0*,*60*
LOS total (days)	6,53 ± *4*,*34*	11,52 ± *6*,*06*	5,30 ± *1*,*19*
LOS peripheral ward (days)	6,53 ± *4*,*34*	11,30 ± *5*,*99*	5,30 ± *1*,*19*
LOS ICU (days)	0,00 ± *0*,*00*	0,22 ± *0*,*57*	0,00 ± *0*,*00*
Deduction (EUR)	0,00 ± *0*,*00*	54,37 ± *186*,*02*	0,00 ± *0*,*00*
Surcharge (EUR)	95,09 ± *339*,*85*	219,76 ± *701*,*76*	0,00 ± *0*,*00*
Effective CW	1,39 ± *0*,*09*	2,41 ± *0*,*20*	2,35 ± *0*,*01*
Effective DRG remuneration (EUR)	5.225,66 ± *354*,*49*	9.049,79 ± *736*,*82*	8.694,73 ± *166*,*87*
Nursing care remuneration (EUR)	1.437,24 ± *1.275*,*60*	2.279,71 ± *1.256*,*18*	824,81 ± *359*,*01*
Additional charge (EUR)	0,00 ± *0*,*00*	1.345,00 ± *641*,*21*	0,00 ± *0*,*00*
Total remuneration (EUR)	6.662,90 ± *1.499*,*41*	12.675,07 ± *1.595*,*22*	9.519,54 ± *455*,*07*
Base rate Bavaria (EUR)	3.771,67 ± *46*,*81*	3.757,04 ± *71*,*91*	3.693,06 ± *53*,*89*
Nursing care fee value (EUR)	275,22 ± *111*,*61*	256,93 ± *116*,*84*	191,77 ± *64*,*03*
OR time (minutes)	100,63 ± *38*,*92*	95,48 ± *23*,*94*	98,00 ± *22*,*74*
Implant costs (EUR)	724,35 ± *75*,*77*	3.470,25 ± *227*,*19*	3.102,84 ± *168*,*72*

*DRG* diagnosis related groups; *EUR* Euro; *ICU* intensive care unit; *LOS* length of stay; *OR* operation; *PCCL* patient clinical complexity level; *SD* standard deviation; *CW* cost weight

### Ratios

Table 3 provides an overview of the DRG reimbursements in relation to LOS, OR time and impant costs as ratios of all groups.

The DRG reimbursement per day of LOS was highest for elective rTSA with 1.641 EUR/day and lowest for nail fixation of proximal femoral fractures with 520 EUR/day.

Regarding OR time, proximal femoral fractures with nail fixation showed the highest DRG reimbursement of 111 EUR/minute. It was lowest for locking plate fixation at the proximal humerus with 52 EUR/minute.

In relation to implant costs, the DRG reimbursement of 15 EUR/EUR was highest for proximal femoral fractures with nail fixation and lowest for rTSA (trauma and elective) with 3 EUR/EUR.

**Table 3:** Ratios of hip and shoulder interventions

Surgical intervention		Ratio	
**Hip**	**DRG remuneration/** **LOS** **(EUR/day)**	**DRG remuneration/** **OR time** **(EUR/minute)**	**DRG remuneration/** **Implant costs (EUR/EUR)**
Nail fixation	519,87	111,01	14,84
Cemented HA	538,47	79,62	9,26
Cementless TA	722,66	76,75	3,88
**Shoulder**	**DRG remuneration/** **LOS** **(EUR/day)**	**DRG remuneration/** **OR time** **(EUR/minute)**	**DRG remuneration/** **Implant costs (EUR/EUR)**
Locking plate fixation	800,25	51,93	7,21
rTSA (Trauma)	785,57	94,78	2,61
rTSA (Elective)	1.640,51	88,72	2,80

*DRG* Diagnosis related groups; *EUR* Euro; *HA* hemi-arthroplasty; *LOS* length of stay; *OR* operation; *rTSA* reverse total shoulder arthroplasty; *TA* total arthroplasty

### Cost data

The highest OR personnel costs of 630 EUR were calculated for rTSA in trauma cases, the lowest costs of 287 EUR for nail fixation at the proximal femur (Table 4).

**Table 4:** Calculation OR personnel costs of hip and shoulder interventions

Hip	Personnel costs OR (EUR) (Mean ± SD)
Nail fixation	286,97 ± *114*,*95*
Cemented HA	498,20 ± *129*,*14*
Cementless TA	449,63 ± *128*,*41*
**Shoulder**	**Personnel costs OR (EUR)** **(Mean ± SD)**
Locking plate fixation	625,97 ± *281*,*50*
rTSA (Trauma)	629,64 ± *190*,*91*
rTSA (Elective)	619,56 ± *229*,*57*

*EUR* Euro; *HA* hemi-arthroplasty; *OR* operation; *rTSA* reverse total shoulder arthroplasty; *SD standard deviation; TA* total arthroplasty

The total OR costs were highest for fracture rTSA with 4.100 EUR and lowest with 685 EUR for nail fixation of proximal femoral fractures (Table 5).

**Table 5:** Calculation OR costs of hip and shoulder interventions

Surgical intervention		OR costs	
**Hip**	**Implant costs (EUR)**	**Personnel costs** **OR (EUR)**	**Total costs (EUR)**
Nail fixation	397,88	286,97	684,85
Cemented HA	748,71	498,20	1.246,91
Cementless TA	1.489,09	449,63	1.938,72
**Shoulder**	**Implant costs (EUR)**	**Personnel costs** **OR (EUR)**	**Total costs (EUR)**
Locking plate fixation	724,35	625,97	1.350,32
rTSA (Trauma)	3.470,25	629,64	4.099,89
rTSA (Elective)	3.102,84	619,56	3.722,40

*EUR* Euro; *HA* hemi-arthroplasty; *OR* operation; *rTSA* reverse total shoulder arthroplasty; *TA* total arthroplasty

For the ward, the highest total personnel costs of 2.520 EUR were calculated for cemented HA, the lowest costs of 958 EUR for elective rTSA (Table 6).

**Table 6:** Calculation personnel costs peripheral ward, ICU and total (peripheral ward + ICU) of hip and shoulder interventions

Hip	Personnel costs Peripheral ward (EUR) (Mean ± SD)	Personnel costs ICU (EUR) (Mean ± SD)	Personnel costs total (EUR) (Mean ± SD)
Nail fixation	2.046,74 ± *1.021*,*44*	138,28 ± *599*,*87*	2.185,02 ± *1.319*,*58*
Cemented HA	2.298,99 ± *959*,*36*	221,42 ± *987*,*28*	2.520,41 ± *1.470*,*72*
Cementless TA	1.461,72 ± *508*,*46*	0,00 ± *0*,*00*	1.461,72 ± *508*,*46*
**Shoulder**	**Personnel costs** **Peripheral ward (EUR)** **(Mean ± SD)**	**Personnnel costs** **ICU (EUR)** **(Mean ± SD)**	**Personnel costs total (EUR)** **(Mean ± SD)**
Locking plate fixation	1.198,12 ± *800*,*61*	0,00 ± *0*,*00*	1.198,12 ± *800*,*61*
rTSA (Trauma)	2.065,90 ± *1.089*,*06*	170,58 ± *436*,*90*	2.236,48 ± *1.208*,*50*
rTSA (Elective)	958,06 ± *214*,*58*	0,00 ± *0*,*00*	958,06 ± *214*,*58*

*EUR* Euro; *HA* hemi-arthroplasty; *ICU* Intensive care unit; *rTSA* reverse total shoulder arthroplasty; *SD standard deviation; TA* total arthroplasty

### Contribution margins

**Fig. 7:**
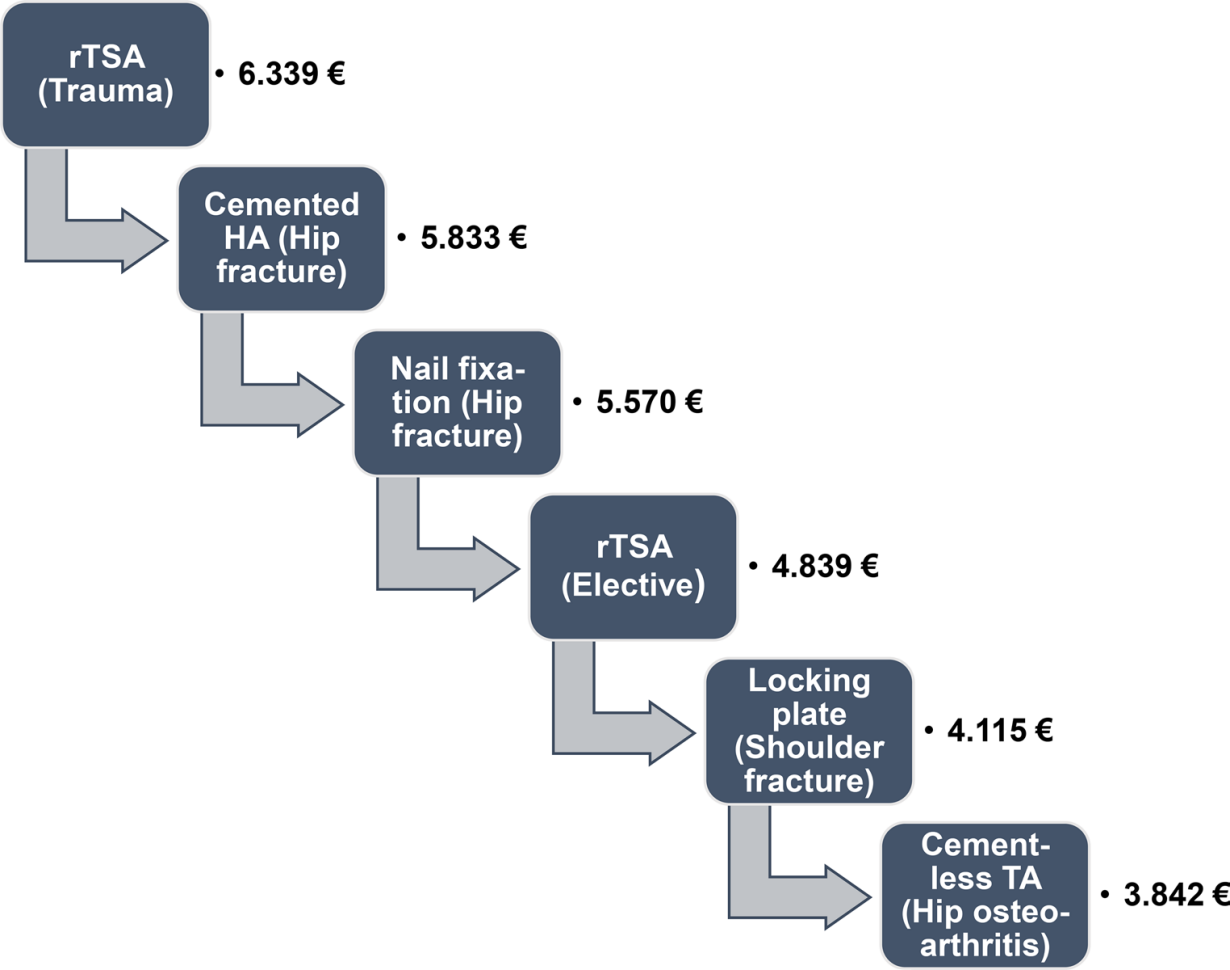
Contribution margins of hip and shoulder interventions. € = EUR

Fracture arthroplasty showed the highest contribution margins with 6.339 EUR for rTSA and 5.833 EUR for HA. It was lowest for TA of the hip with 3.842 EUR (Fig. 7).

## Discussion

This study was designed to analyze DRG reimbursements of elective total arthroplasty of hip and shoulder joint compared to acute fracture interventions with regard to LOS, OR time, implant and personnel costs.

Overall, fracture arthroplasty of the hip and shoulder showed the highest DRG remuneration and estimated contribution margins with 6.930 EUR and 5.833 EUR respectively for cemented HA of femoral neck fractures as well as 9.050 EUR and 6.339 EUR respectively for rTSA of proximal humeral fractures.

However, it should first be noted critically for the classification of the results that in our study, more complex cases with revenue-relevant secondary diagnoses and higher-value DRGs were excluded in all groups to allow better comparability of acute trauma patients and elective osteoarthritis patients. The exclusion of these cases may lead to an overestimation of the economic performance of acute fracture interventions. Since more complex trauma cases with higher PCCL values and DRG revenues, but also very high overall treatment costs, account for a significant portion of daily trauma care, our results do not comprehensively reflect clinical practice.

At the hip, the ratios DRG reimbursement/OR time of 111 EUR/minute and DRG reimbursement/implant costs of 15 EUR/EUR were highest for nail fixation of proximal femoral fractures followed by HA with 79 EUR/minute and 9 EUR/EUR respectively. In fracture scenarios, nail osteosynthesis seems to be better reimbursed due to the lower implant costs and shorter OR time compared to arthroplasty. The cost analysis of Liu et al. showed comparable results with a higher cost-effectiveness of internal fixation for femoral neck fractures compared to hemi-arthroplasty, since total costs were lower [13, 14].

Our results showed that implant costs for hip and shoulder arthroplasty are much higher than for osteosynthetic treatments. The DRG reimbursement regarding implant costs was lowest for fracture and elective rTSA at the shoulder with 3 EUR/EUR and second lowest for elective TA at the hip with 4 EUR/EUR. Implants represent a major cost driver in arthroplasty, with prices varying considerably internationally in recent years [1, 15]. Particularly in revision arthroplasty, certain complex procedures cannot be performed cost-effectively due to the high implant costs, as demonstrated by a cost-benefit analysis by Awwad et al. using mega-prostheses [16].

Reverse shoulder arthroplasty is considered a cost-intensive procedure for the treatment of proximal humeral fractures due to the high costs of prostheses. This is demonstrated by a study by Rosas et al., who found in a cost analysis of three standard surgical procedures for proximal humeral fractures, that rTSA had the highest initial and total treatment costs compared to hemiarthroplasty and plate osteosynthesis [17]. Packer et al. analyzed the treatment costs of rTSA for proximal humeral fractures and showed that implant and personnel costs are the primary cost drivers, with implants accounting for up to 40% of the total costs [18]. However, Politzer et al. concluded that despite the higher initial costs, reverse shoulder arthroplasty should be considered for the treatment of complex proximal humeral fractures, as it has similarly high total costs within the first postoperative year as hemi-arthroplasty [19]. Further studies have shown that reverse shoulder replacement can be a cost-effective strategy for fracture treatment in the long term compared to plate osteosynthesis [20–23].

Despite the high implant costs, elective rTSA is also considered a cost-effective arthroplasty procedure, as Bachman et al. demonstrated in a prospective study comparing reverse shoulder arthroplasty and total hip arthroplasty in osteoarthritis with regard to cost-effectiveness and quality of life [24]. Coe et al. and Cregar et al. found similar conclusions by cost-effectiveness analyses of elective reverse shoulder arthroplasty [25, 26].

Generally, surgical costs are largely influenced not only by implant costs but also by personnel costs, which depend on the OR time and the professional qualifications of the staff. In our study, we calculated the highest overall surgical costs of 4.100 EUR for rTSA in fracture scenarios due to the highest implant and personnel costs. The lowest total surgical costs of 685 EUR we found for nail fixation of proximal femoral fractures based on the lowest implant costs and the shortest OR time, which further underlines the results mentioned above.

In terms of length of stay in the hospital, elective hip and shoulder arthroplasty showed the highest DRG reimbursements. For elective total hip arthroplasty, the DRG remuneration per day was 723 EUR with a mean LOS of 8 days. Since patients with proximal femoral fractures had a LOS up to 5 days longer, the DRG reimbursement for cemented HA and nail osteosynthesis was approximately 200 EUR per day lower. Compared to elective hip patients, patients with proximal femoral fractures are usually older with many comorbidities and longer lengths of stay, often in specialized geriatric trauma centers, which can lead to higher treatment costs and revenue deficits for the hospitals [27, 28]. This is demonstrated by the results of Rohrer et al., who found a correlation between longer LOS for multimorbid orthopaedic patients and higher costs as well as revenue losses in the Swiss DRG system [4].

For the shoulder, in our study the DRG remuneration per day was even 1.641 EUR for elective rTSA with a mean length of stay of five days versus 786 EUR for rTSA in trauma cases with a much longer LOS of 12 days.

This confirms the data of Menendez et al., who found a correlation between total treatment costs and length of stay, but not with the operating time for reverse shoulder replacement [29]. In addition, Rosas et al. showed by retrospective cost analysis of shoulder arthroplasty that revenues are significantly influenced by comorbidities, which affect length of stay [30].

In the University Orthopaedic Trauma Department examined in the present study, both acute trauma patients and elective osteoarthritis patients are mobilized postoperatively through physiotherapy, and discharge goals are defined depending on the general condition, home care situation and mobility status, including transfer to an orthopedic or geriatric rehabilitation clinic or discharge home. For geriatric, frail patients, there is also a structured concept involving geriatricians and acute geriatric early complex treatment by an interdisciplinary team of specialized medical and nursing service and therapists within the framework of a certified geriatric trauma center. The management of postoperative care and mobilization generally has a significant structural influence on the length of hospital stay and thus on treatment costs, and should therefore be designed as efficiently as possible, taking into account the patients’ clinical condition.

Due to the longer LOS of patients with proximal femoral and proximal humeral fractures, the calculated total personnel costs for the ward in our study were highest with 2.520 EUR and 2.236 EUR compared to patients undergoing elective hip and shoulder arthroplasty with 1.462 EUR and 958 EUR respectively.

Despite the higher total ward personnel costs, our results showed higher estimated contribution margins for acute fracture interventions compared to elective arthroplasty procedures, especially at the hip. However, these are merely case-specific, approximately calculated contribution margins, which ultimately do not allow any conclusions about the profitability of the department. Since structural costs, such as emergency department infrastructure or the standby capacity of the operating room and intensive care unit, were not considered in the calculations in the present study, the significance of the results regarding the overall structural profitability of the clinic is limited.

To summarize, the classification of trauma cases with fractures into higher-rated DRG case-based flat rates is appropriate and justified, especially in older multimorbid patients with longer length of stay and costly inpatient treatment. Despite trauma cases seem to be better reimbursed, there is a considerable higher effort with 24-hour availability of emergency room and operating room in trauma surgery compared to elective orthopaedics, which can lead to a financial burden for the hospitals [31]. Furthermore, the above-mentioned fact that treatment of acutely injured and more fragile patients is most likely more expensive compared to elective orthopaedic patients justifies higher reimbursements in trauma and seems to be appreciated by the aG-DRG system.

### Limitations

This retrospective study is limited by several factors. First, there was no financial analysis of total hospital costs possible due to the structure of cost recording at the University Hospital with missing data of total treatment costs per case. Therefore, personnel costs were calculated by model and contribution margins were estimated. An assessment of the cost coverage rate from the hospital’s perspective was not possible. Another limitation is that personnel and implant costs can vary between hospitals, influencing the total treatment costs and the cost coverage rate. Furthermore, this study is certainly limited regarding generalizability, as the results are not universally applicable and the estimated contribution margins are institution-specific outputs, not system-level truths. Another limitation is the purely descriptive statistical analysis without formal statistical comparisons, which nevertheless allowed the extensive data set to be presented in an overview in terms of a health economic revenue analysis.

## Conclusion

Elective total arthroplasty of the hip and shoulder joint is not generally more cost-effective in the current aG-DRG system. Acute fracture-related surgical interventions at the proximal femur and the proximal humerus seem to be better reimbursed regarding OR time and implant costs, elective total arthoplasty seems more profitable regarding LOS in the hospital. Due to the high standby costs for the 24-hour availability of emergency room, intensive care unit and operating room capacity, traumatology can represent a financial burden for the hospitals. The treatment of acutely injured and often multimorbid patients is usually more complex and costly compared to elective orthopaedic patients. Therefore, a performance-based representation of treatments within the aG-DRG system and the introduction of availability payment for traumatology planned as part of the German hospital reform appear justified in order to reduce the economic pressure on hospitals.

## Data Availability

The datasets generated and analyzed during the current study are available from the corresponding author upon reasonable request. Data are located in controlled access data storage at the University Hospital Regensburg.
